# Magnetite Nanoparticles and Carbon Nanotubes for Improving the Operation of Mesophilic Anaerobic Digesters

**DOI:** 10.3390/microorganisms11040938

**Published:** 2023-04-03

**Authors:** Elvira E. Ziganshina, Ayrat M. Ziganshin

**Affiliations:** Department of Microbiology, Institute of Fundamental Medicine and Biology, Kazan (Volga Region) Federal University, 420008 Kazan, Russia

**Keywords:** anaerobic digestion, chicken manure, conductive materials, nanomagnetite, carbon nanotubes, volatile fatty acids, bacterial community, methanogenic community

## Abstract

Anaerobic waste processing contributes to the development of the bioenergy sector and solves environmental problems. To date, many technologies have been developed for increasing the rate of the anaerobic digestion process and yield of methane. However, new technological advancements are required to eliminate biogas production inefficiencies. The performance of anaerobic digesters can be improved by adding conductive materials. In this study, the effects of the separate and shared use of magnetite nanoparticles and carbon nanotubes in anaerobic digesters converting high-nitrogen-containing waste, chicken manure, were investigated. The tested nanomaterials accelerated the methane production and increased the decomposition of products from the acidogenesis and acetogenesis stages. The combined use of magnetite nanoparticles and carbon nanotubes gave better results compared to using them alone or without them. Members of the bacterial classes *Bacteroidia*, *Clostridia*, and *Actinobacteria* were detected at higher levels in the anaerobic digesters, but in different proportions depending on the experiment. Representatives of the genera *Methanosarcina*, *Methanobacterium*, and *Methanothrix* were mainly detected within the methanogenic communities in the anaerobic digesters. The present study provides new data for supporting the anaerobic treatment of substrates with a high content of inhibitory compounds, such as chicken wastes.

## 1. Introduction

Anaerobic digestion (AD) is one of the most widely employed biological processes for biowaste management and biogas production (a mixture of mainly methane and carbon dioxide). Continuous research in the field of biogas production is associated with the need to dispose of huge masses of waste and the demand for the use of renewable alternative resources in the face of limited reserves of traditional fossil raw materials [1]. Municipal waste, industrial residues and by-products, and agricultural substrates can be used to produce biogas. Agro-industrial wastes are one of the important sources for the production of biogas [2]. Biogas, which has a wide range of applications (as a fuel for transport, a source of heat, and a source of electricity), increases the sustainability of agriculture by reducing pollutant emissions [3].

Biomass under anaerobic conditions is converted by complex microbial consortiums to biogas and a nutrient-rich digestate, which can then be used as agricultural fertilizer [4,5] and a nutrient media for microalgal cultivation [6,7,8]. Anaerobic microorganisms are involved in four main processes of biogas production: hydrolysis, acidogenesis, acetogenesis (bacterial stages), and methanogenesis (the final stage carried out by methanogenic archaea) [9]. During the degradation of organic materials, different and specialized bacteria produce intermediates for methanogens, which keep these intermediates at low levels. Methanogenic archaea perform methanogenesis through three pathways: hydrogenotrophic (performed by most methanogens), acetoclastic (performed by *Methanothrix* and *Methanosarcina*), and methylotrophic [10]. The syntrophic relationship between bacteria and methanogens is important for biogas production through AD. Interspecies electron transfer (IET) via hydrogen or formate is an important mechanism for electron transfer between syntrophic bacteria that oxidize volatile fatty acids (VFAs) and methanogens that produce methane. Direct interspecies electron transfer (DIET) is noted as a breakthrough in understanding the function of methanogenic communities and is considered an alternative pathway occurring through conductive pili or *c*-type cytochromes, as well as different conductive materials. The rate of such transfer from bacteria to methanogens is higher [11,12].

Nanomaterials are attractive additives in AD and are used to enhance microbial activity in various anaerobic systems [13,14,15,16,17,18]. Such additives as iron-based nanomaterials (e.g., nanomagnetite (Fe_3_O_4_)), carbon nanotubes (CNTs), and nanoscale zero-valent metals are tested to enhance anaerobic processes at high organic loading rates [19], during treatment of high-solid substrates [15,20], and under ammonia-stressed conditions [16,21,22]. CNTs are noted as potential additives for the anaerobic digestion of various substrates due to their conductivity and adsorption properties [14,18,23]. Nanomagnetite is considered an electrically conductive material that enhances the DIET pathway and helps reduce the inhibition of anaerobic microbial communities by high levels of ammonia [16]. Wang et al. [15] found that the addition of nanomagnetite enhanced electron transfer efficiency, decreased the concentration of VFAs, and improved the methane yield from sewage sludge. In the study performed by Di et al. [22], the addition of nano-Fe_3_O_4_ biochar increased the methane production from chicken manure by 63%. Nevertheless, the influence of conductive materials separately and jointly has not been fully studied, and the mechanism of their action in anaerobic systems with a high concentration of inhibitory substances is subject to verification by additional experiments.

Chicken manure has a relatively high energy content, but anaerobic conversion of this substrate has high risks of reduction of the process or inhibition due to the high content of ammonia formed during the hydrolysis of protein and uric acid [24,25]. To improve the anaerobic digestion of chicken manure, processes such as co-digestion [20], ammonia adsorption on zeolites [26], and supplementation of reactors with conductive materials have been extensively studied [22,27]. Based on the known beneficial effects of Fe_3_O_4_- and CNTs-mediated stimulation of methanogenesis and our previous scientific results, we hypothesize that co-supplementation of biogas reactors with nanomagnetite and carbon nanotubes may attenuate the inhibitory effect of ammonia on methanogenesis and provide more electron transfer sites, thus enhancing specific microbial interactions in anaerobic systems converting chicken waste.

In the present study, the influence of magnetite nanoparticles and carbon nanotubes on biomethane production from agricultural waste (chicken manure) is studied in detail. Additionally, the bacterial 16S rRNA gene and the methyl coenzyme-M reductase a-subunit (*mcrA*) gene are further characterized to clarify various functional groups of microorganisms in mesophilic anaerobic digesters without and with the combined use of nano-Fe_3_O_4_ and CNTs.

## 2. Materials and Methods

### 2.1. Biowaste and Inoculum

Chicken manure with total solids (TS) of 59.4 ± 0.7% and volatile solids (VS) of 52.2 ± 0.5% was used as substrate for the anaerobic digestion tests. Chicken manure was obtained from a local chicken farm (Kazan, Republic of Tatarstan, Russia). The microbial consortium of anaerobically digested cattle manure was used as an inoculum in the anaerobic process. Cattle manure was obtained from a local dairy farm (Kazan, Republic of Tatarstan, Russia).

### 2.2. Additives

Magnetite nanoparticles (Fe_3_O_4_, 50–100 nm particle size, Sigma–Aldrich, St. Louis, MI, USA) and hydrophilic (“soluble” in water (up to 0.2%)) multi-walled carbon nanotubes of the “Taunit” series were used as additives. Carbon nanotubes were quasi-one-dimensional, nanoscale, filamentous polycrystalline graphite cylindrical formations with internal channels in the form of a black powder, had a hollow cylindrical structure, at least 2 µm long, with an external diameter of 10–30 nm and an internal diameter of 5–15 nm. “Taunit-M” carbon nanotubes were produced by chemical vapor deposition; nanocarbon content: ≥95 wt/% (available online: http://eng.nanotc.ru/producrions/87-cnm-taunit; accessed on 25 March 2023).

### 2.3. Biochemical Methane Potential Experiments and Analytical Methods

The biochemical methane potential of chicken manure was estimated using Automatic Methane Potential Test Systems (AMPTS II Light, Bioprocess Control, Lund, Sweden). All batch anaerobic reactors were started using an inoculum (digested cattle manure) and chicken manure as a substrate. The inoculum to substrate ratio was 34.8/52.2 g of VS (TS concentration of 6.5%) for the batch tests. The biogas reactors with a working volume of 1.6 L were incubated at 38 °C for 30 days. Nano-Fe_3_O_4_ and CNTs at concentrations of 3.2 g L^−1^ and 3.5 g L^−1^ were selected and separately added to the experimental reactors (R_M and R_CNT, respectively). In the reactors R_M_CNT, Fe_3_O_4_ and CNTs were added jointly at the same concentrations. Control reactors operated without the addition of any additives. Blank reactors (with only inoculum) were also used to compensate for the CH_4_ level produced by the inoculum itself. The biogas was initially passed through a solution of 3 M NaOH to remove CO_2_ and H_2_S, and the CH_4_ yield was estimated with a gas flow meter system. The AMPTS II Light instruments agitated the digestion medium for 1 min at 60 rpm, followed by a 3 min rest interval.

CH_4_ values were obtained automatically from the AMPTS II instruments and normalized to 1.0 standard atmospheric pressure, 0 °C, and zero moisture content. The option of compensating for the volume of gas initially used for flushing the system was selected to avoid overestimation of gas volume and gas flow. Samples were periodically taken from reactors for various analyses, including volatile fatty acids and total ammonia nitrogen (TAN) concentrations. These analyses were performed as detailed by us previously [18,28,29]. Each test was conducted in duplicate, all analyses were measured in triplicate, and the mean values are presented together with standard deviations. The Tukey method and 95% confidence were used to compare differences (Minitab software version 20.2.0, State College, PA, USA).

### 2.4. Microbial Community Structure Analysis

Samples for bacterial and archaeal community analysis were collected on days 6 and 13 of the experiment and immediately processed. The microbial community of each sample was analyzed using high-throughput 16S rRNA and *mcrA* genes sequencing. DNA was extracted using the FastDNA Spin Kit for Soil (MP Biomedicals, CA, USA). Bacterial 16S rRNA gene fragments were amplified via polymerase chain reaction using primers Bakt_341F (5′-CCT ACG GGN GGC WGC AG-3′) and Bakt_805R (5′-GAC TAC HVG GGT ATC TAA TCC-3′). Mlas (5′-GGT GGT GTM GGD TTC ACM CAR TA-3′) and mcrA-rev (5′-CGT TCA TBG CGT AGT TVG GRT AGT-3′) primers were used to amplify the *mcrA* gene of methanogenic archaea. The amplicons were purified using the QIAquick PCR Purification Kit (QIAGEN, Germany). High-throughput amplicon sequencing was performed on the Illumina MiSeq platform (Illumina Inc., San Diego, CA, USA), and data analysis was performed as detailed by us previously [28,29,30].

## 3. Results and Discussion

### 3.1. Process Stability and Methane Production

During the experiments, four different conditions were monitored: control reactors (R_C), reactors supplemented with Fe_3_O_4_ nanopowder (R_M), reactors supplemented with CNTs (R_CNT), and reactors supplemented jointly with Fe_3_O_4_ nanopowder and CNTs (R_M_CNT). According to our previous experiments on variations in total CH_4_ production and CH_4_ flow rate influenced by different concentrations of the selected additives, 3.2 g L^−1^ of nano-Fe_3_O_4_ and 3.5 g L^−1^ of CNTs were chosen as the optimum concentrations in this study, considering the stimulating effect and further practical applications.

**Figure 1:**
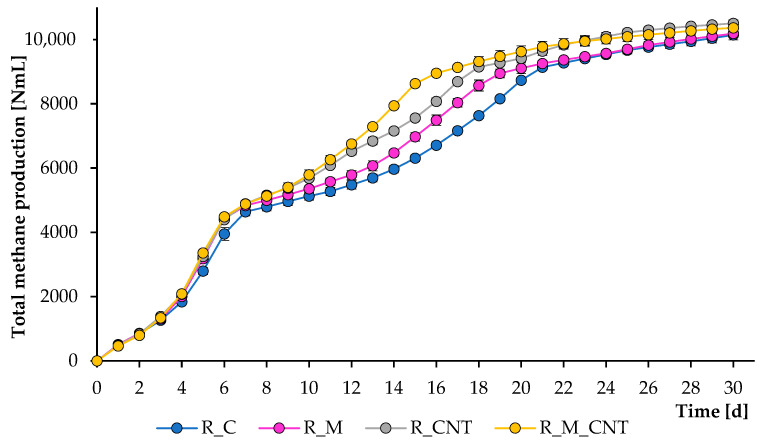
Influence of magnetite nanoparticles and carbon nanotubes on total methane production during the anaerobic digestion of chicken manure. Error bars represent the standard deviation of duplicate tests.

**Figure 2:**
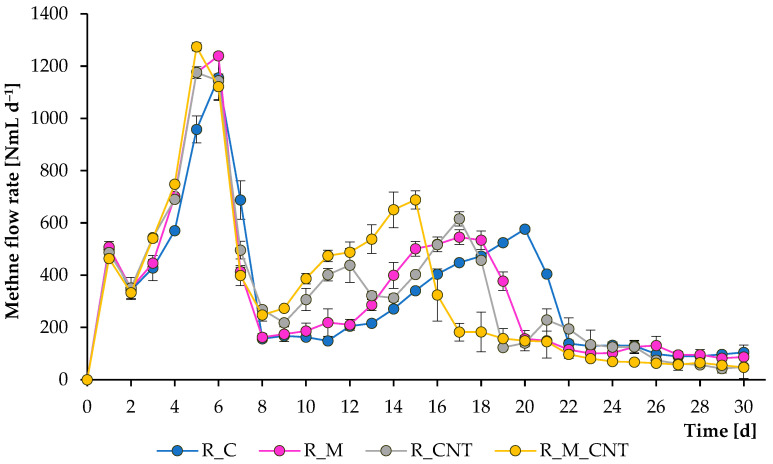
Influence of magnetite nanoparticles and carbon nanotubes on methane flow rate during the anaerobic digestion of chicken manure. Error bars represent the standard deviation of duplicate tests.

The operation of the mesophilic reactors continued for 30 days, and during this period, several samples were removed from them to analyze the composition of the digesting mixture. Figure 1 and Figure 2 illustrate the total CH_4_ production and CH_4_ flow rate, respectively. As can be seen, CH_4_ was formed effectively in all experiments; however, adding 3.2 g L^−1^ of nano-Fe_3_O_4_ and 3.5 g L^−1^ of CNTs could substantially accelerate the methanogenesis stage, and the joint application of both supplements improved the anaerobic process more efficiently. The reactors produced CH_4_ immediately after the addition of the chicken manure. During the first week of experiments, more CH_4_ was produced from the reactors supplemented with either nano-Fe_3_O_4_ or CNTs, and no significant changes were observed between the treatments with additives, which may be related to the effective initial transformation of soluble organic matter.

However, further experiments demonstrated that all additives significantly improved the rate of CH_4_ formation, and the greatest positive effect was observed when nano-Fe_3_O_4_ and CNTs were used jointly. If the first large peak of CH_4_ formation in all reactors occurred on days 5–6, then the last peak in the reactors with the simultaneous introduction of agents was noted on days 15–17, which is earlier than the 3–5 days of the control group. These latter peaks were almost certainly caused by microbial decomposition of less degradable compounds. The final total CH_4_ yield levels (at day 30) for the reactors R_C, R_M, R_CNT, and R_M_CNT were 10,144 ± 153 mL, 10,187 ± 46 mL, 10,504 ± 106 mL, and 10,363 ± 113 mL, respectively. The final specific CH_4_ production values (at day 30) for the reactors R_C, R_M, R_CNT, and R_M_CNT were 194 ± 2.9 mL g^−1^_VS_, 196 ± 1.0 mL g^−1^_VS_, 201 ± 2.0 mL g^−1^_VS_, and 199 ± 2.2 mL g^−1^_VS_, respectively. These results indicate that the presence of nano-Fe_3_O_4_ or CNTs (both separately and jointly) increases the methane production rate from chicken manure.

**Figure 3:**
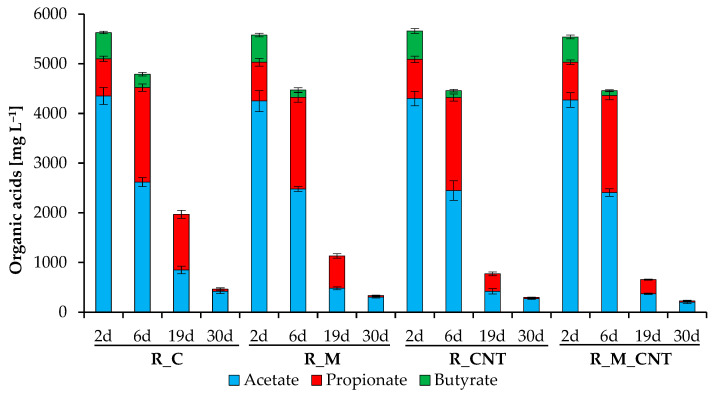
Influence of magnetite nanoparticles and carbon nanotubes on individual organic acid concentrations during the anaerobic digestion of chicken manure. Error bars represent the standard deviation of duplicate tests.

Figure 3 shows the concentrations of the individual volatile fatty acids (acetic acid, propionic acid, and n/iso-butyric acids). In all reactors, approximately the same total level of theses volatile fatty acids was initially determined, with acetic acid and propionic acid being the dominant types of VFAs. The supplementation of reactors with nano-Fe_3_O_4_ and CNTs promoted earlier consumption of acetate and butyrate, and the joint addition of nano-Fe_3_O_4_ and CNTs stimulated the more effective involvement of these VFAs in methanogenesis.

In contrast, the biodegradation of propionate occurred after about one week of the experiments, with the appearance of the last peak of CH_4_ yield. This indicates that propionate-utilizing microorganisms were activated after a decrease in the level of acetate and almost complete utilization of butyrate, and the use of additives stimulated their activity. Finally, the joint addition of nano-Fe_3_O_4_/CNTs, separate addition of CNTs, and finally separate addition of nano-Fe_3_O_4_ into anaerobic reactors promoted the uptake of the formed VFAs. The obtained results are consistent with previous research that found acetate, propionate, and butyrate to be more rapidly biodegradable in the presence of magnetite, carbon nanotubes, and several other conductive materials (but these results were obtained during the anaerobic digestion of other substrates) [11,15,18,22,31].

**Figure 4:**
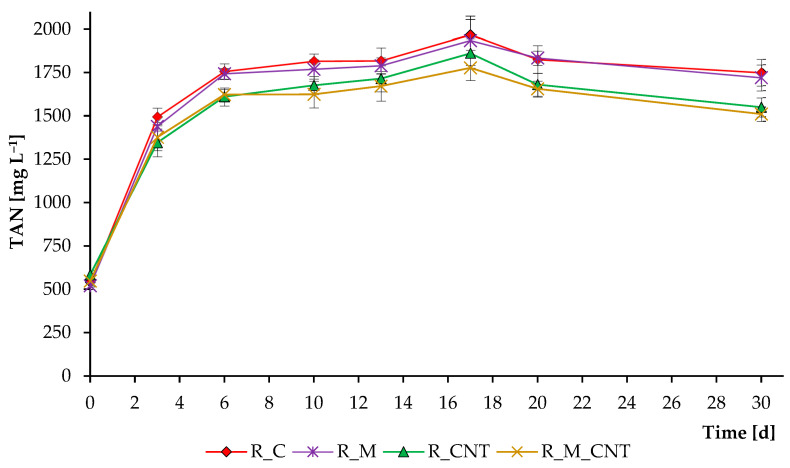
Influence of magnetite nanoparticles and carbon nanotubes on total ammonia nitrogen concentrations during the anaerobic digestion of chicken manure. Error bars represent the standard deviation of duplicate tests.

Figure 4 demonstrates the total ammonia nitrogen concentration over the course of the whole anaerobic digestion process. Decomposition of the high amount of nitrogenous organic matter led to an increase in TAN level in all reactors. Interestingly, reactors supplemented with CNTs resulted in a lower level of TAN. The higher microbial activity in the reactors supplemented with CNTs could stimulate the enhanced assimilation of ammonia by microbial cells. In addition, CNTs have different adsorption properties that allow them to actively bind to other molecules [23,32]. Therefore, ammonia and other N-containing compounds (from which ammonia was formed) could bind to the tested carbon nanotubes of the “Taunit-M” series.

**Figure 5:**
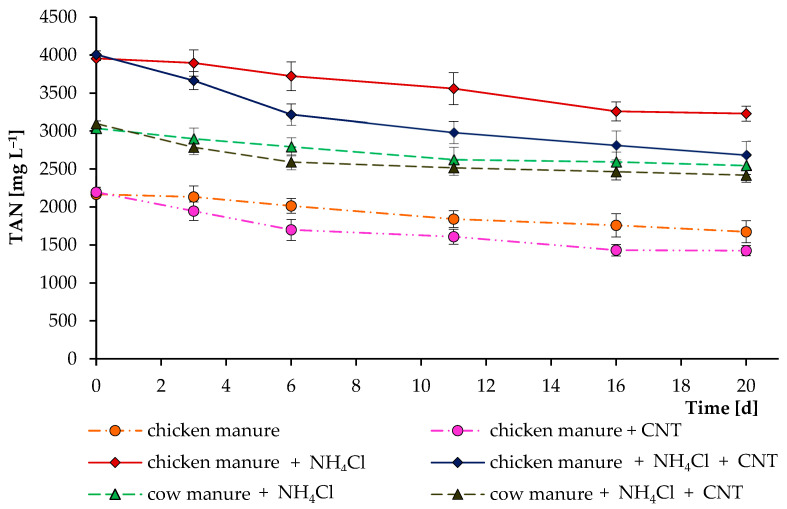
Influence of carbon nanotubes on total ammonia nitrogen concentrations during their incubations in digested chicken and cow manures. In separate experiments, ammonium chloride was added to increase the initial level of total ammonia nitrogen. Error bars represent the standard deviation (n = 4).

To test this assumption, additional short incubation experiments with the digested cow and chicken manures were performed (Figure 5). These experiments clearly demonstrated that CNTs can actively remove TAN from the digesting mixture, reducing toxicity and possibly increasing the activity of anaerobic microorganisms. We also performed experiments on the addition of (NH_4_)_2_SO_4_ and NH_4_Cl as sources of NH_4_^+^ (1–2 g L^−1^) to a sterilized K-Na-phosphate buffer (pH 7 and 8). After adding 2–5 g L^−1^ of CNTs and 3 weeks of experiments, no significant changes in the level of NH_4_^+^-N were observed. This denotes a specific TAN removal in specific environments. Another study found that the addition of CNTs could either mitigate or worsen ammonia inhibition, depending on the concentration of TAN in the system [33].

Carbon nanotubes and iron-based nanomaterials have been noted by many scientific groups as useful agents for stimulating the anaerobic digestion process, especially in the conversion of complex substrates [15,21,22,33,34]. In the present work, their positive joint effect on methanogenesis was noted. Researchers in this field attribute the stimulation of methanogenesis by these materials to many factors. The stimulation of methanogenesis by the addition of such additives mainly depends on their conductivity and adsorption properties, as well as on the response of the microbial community, which generally improves the production of CH_4_.

Mostafa et al. [35] observed a positive effect of nano-sized magnetite (0.5 g Fe g _VS_^−1^) and carbon nanotubes (1 g L^−1^) on the anaerobic digestion of oleic acid at various concentrations by increasing conductivity and stimulating DIET in the microbial community. Li et al. [36] showed that the introduction of carbon nanotubes up to 1.0 g L^−1^ into the anaerobic process intensified anaerobic wastewater treatment through rapid substrate consumption and increased electrical conductivity of the sludge, which could promote DIET between anaerobic fermentative bacteria and methanogenic archaea. In other studies, anaerobic systems with magnetite [21] and CNTs [33] showed high resistance to inhibition by ammonia.

Finally, nano-sized materials may improve anaerobic digestion performance both through DIET-related mechanisms and through other important mechanisms, such as maintaining a more negative redox potential in anaerobic microcosms [37] or by providing protection for microbes from inhibitory factors [21,33]. In conclusion, given the advantages of the tested materials, the conversion rate and stability of anaerobic systems operated with the combined use of nano-Fe_3_O_4_ and CNTs can be significantly improved in real-scale applications.

### 3.2. Variation in Microbial Communities

To identify the microorganisms responsible for all stages of anaerobic digestion of chicken manure and clarify the effect of the combined use of magnetite nanoparticles and carbon nanotubes on the structure and dynamics of the microbial communities, the taxonomic distribution of bacterial and archaeal communities in the control (R_C) and experimental (R_M_CNT) reactors was determined. Only one replicate from duplicate tests was analyzed on days 6 and 13 of the anaerobic process.

A total of 90,839 high-quality bacterial 16S rRNA gene sequences and 40,897 high-quality *mcrA* gene sequences were obtained. Alpha diversity indices are presented in Table 1. Operational taxonomic units (OTUs) in samples from the reactors were obtained based on a relative abundance more than 0.01%. In both systems (R_C and R_M_CNT), the number of bacterial and methanogenic OTUs, as well as the species richness estimator Chao1, the Shannon’s diversity index, and the Simpson’s evenness estimator, were higher in the second analyzed sample.

Bacterial OTUs were grouped taxonomically from the phylum to the genus level. On the phylum level, there were minor differences between the bacterial communities associated with the anaerobic reactors, with *Firmicutes* (ranging from 32% to 35% of the relative abundance) and *Bacteroidetes* (ranging from 27% to 35%) as the dominant phyla in the reactors R_C and R_M_CNT. The relative abundances of representatives within the phyla *Firmicutes* and *Bacteroidetes* in the R_C were 32% and 34% on day 6 and 35% and 27% on day 13, respectively. The bacterial communities in the R_M_CNT were characterized by representatives assigned also to the major phyla *Firmicutes* (32–35%) and *Bacteroidetes* (34–35%). In addition, members of the phyla *Actinobacteria*, *Proteobacteria*, *Synergistetes*, *Chloroflexi*, *Planctomycetes,* and *Spirochaetes* were also determined in both reactors, but at different levels. According to the literature [17,22,29,38], *Firmicutes* and *Bacteroidetes,* with their involvement in N-rich substrate degradation and fermentation processes, appear to be common phyla in the anaerobic digestion of chicken manure.

**Figure 6:**
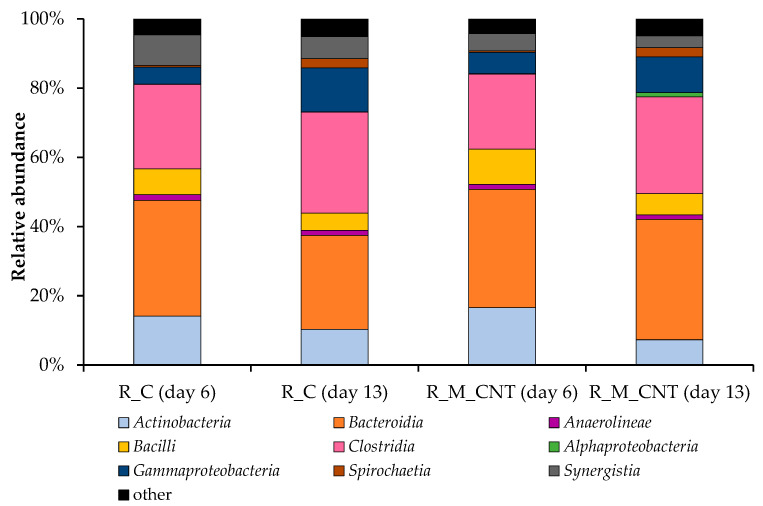
Taxonomic composition of bacterial communities in the anaerobic reactors as determined by amplicon sequencing of bacterial 16S rRNA genes (analyzed on day 6 and day 13). Bacterial community composition is shown on the class level. Classes with abundances below 1.0% are summarized as “other”.

Figure 6 illustrates the classification of bacterial communities on the class level, where *Bacteroidia* and *Clostridia* were the dominant classes in the control reactor R_C and experimental reactor R_M_CNT. Representatives of the class *Bacteroidia* accounted for 34% of the relative abundance in the control reactor on day 6, decreased in this system on day 13 (27%), but were at the same level in the reactor R_M_CNT (34–35%). The joint addition of the tested nanomaterials into the R_M_CNT increased the relative abundance of the representatives of the classes *Actinobacteria*, *Bacilli*, and *Gammaproteobacteria* (on day 6). In contrast, this addition decreased the relative abundance of the species within the classes *Clostridia* and *Synergistia* (on days 6 and 13) in the same reactor compared to the control system. In both anaerobic mesophilic biogas reactors, the relative abundance of *Actinobacteria*, *Bacilli*, and sugar-fermenting bacteria of the class *Synergistia* decreased, whereas the relative abundance of representatives of the bacterial classes *Clostridia*, *Alphaproteobacteria*, *Gammaproteobacteria*, and *Spirochaetia* increased at the middle of the experimental period.

**Figure 7:**
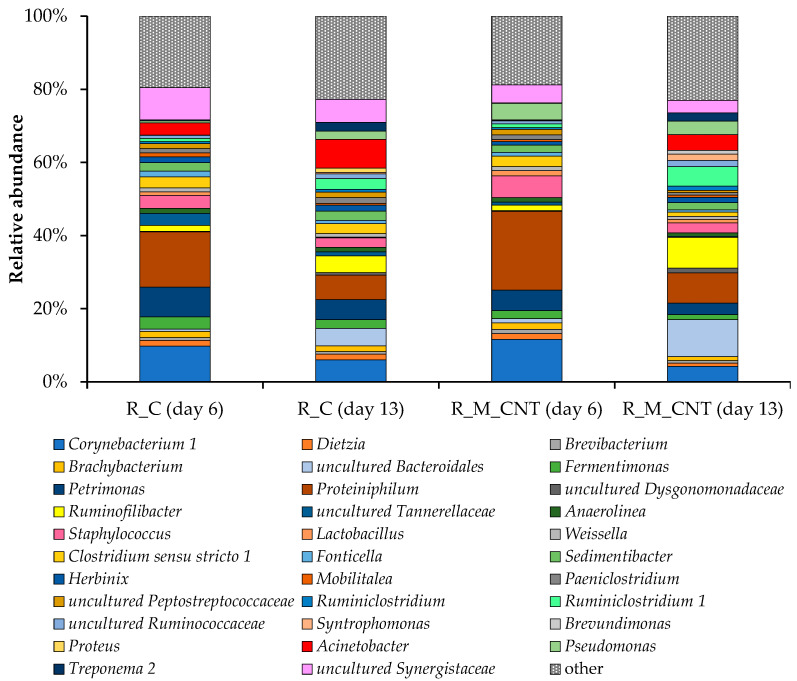
Taxonomic composition of bacterial communities in the anaerobic reactors as determined by amplicon sequencing of bacterial 16S rRNA genes (analyzed on day 6 and day 13). Bacterial community composition is shown on the genus level. Only genera comprising at least 1% relative abundance in at least one sample are presented.

On the genus level, the bacterial communities of the anaerobic reactors with and without additives showed the robustness of the core microbiome. Despite this, there were several differences between the bacterial communities that functioned in the control reactor and the reactor supplemented with additives (Figure 7). Members of the bacterial genera *Proteiniphilum* (15–22%), *Corynebacterium* 1 (10–12%), and *Petrimonas* (6–8%) were the most abundant in samples from both reactors on day 6, while on day 13 of the experiment, uncultured *Bacteroidales* and *Ruminococcaceae*, members of the genera *Ruminofilibacter*, *Ruminiclostridium* 1, and *Acinetobacter* came to important positions. Members of the *Bacteroidales* with metabolic diversity can play important roles in the biodegradation of proteins and amino acids [39]. *Ruminococcaceae* species play important roles in different stages of the anaerobic digestion process, including hydrolysis [40,41] and the production of organic acids, CO_2_, and H_2_ [15,42,43]. This may explain the increase in their proportion in both reactors.

It should be noted that the proportion of *Syntrophomonas*, uncultured *Bacteroidales*, *Ruminiclostridium* 1, *Ruminofilibacter*, *Ruminiclostridium*, and members of the *Ruminococcaceae* increased markedly in the reactor with additives (up to +297%, +112%, +84%, +82%, +60%, and +30%, respectively, on day 13), and distinct bacteria were characterized only for the R_M_CNT, for example members of the genera *Brevundimonas*. The novel unknown genus, which belongs to the order *Bacteroidales*, made up 10% of the relative abundance of the most important bacterial genera in the experimental reactor, while in the control reactor its share was no higher than 5%. Many members of the *Bacteroidales* are involved in the hydrolysis, acidogenesis, and acetogenesis steps of the anaerobic digestion process [44,45]. Another important genus of the order *Bacteroidales*, *Ruminofilibacter*, made up 9% of the major players in the reactor with additives. Members of *Syntrophomonas*, as typical syntrophic bacteria involved in the oxidation of short-chain fatty acids and partners of hydrogen/formate-utilizing microorganisms, have been identified as important in various other anaerobic digestion systems [35,46,47]. Representatives of this genus have been proposed as bacteria that can potentially create magnetite-mediated DIET with several methanogenic archaea [35,48,49]. Viggi et al. [11] noted an improvement in methane production of up to 33% when adding micrometer-sized magnetite to a real anaerobic digestion process. The authors reported that the additive triggered DIET between propionate-oxidizing acetogens and carbon dioxide-reducing methanogens and promoted a fast and less sensitive to external hydrogen partial pressure conversion of propionate to methane. Therefore, an increase in the proportion of known and unknown bacteria had a positive effect on methanogenesis, including acceleration of the hydrolysis rate and conversion of butyrate and propionate to methanogenic precursors (acetate, hydrogen, or formate).

**Figure 8:**
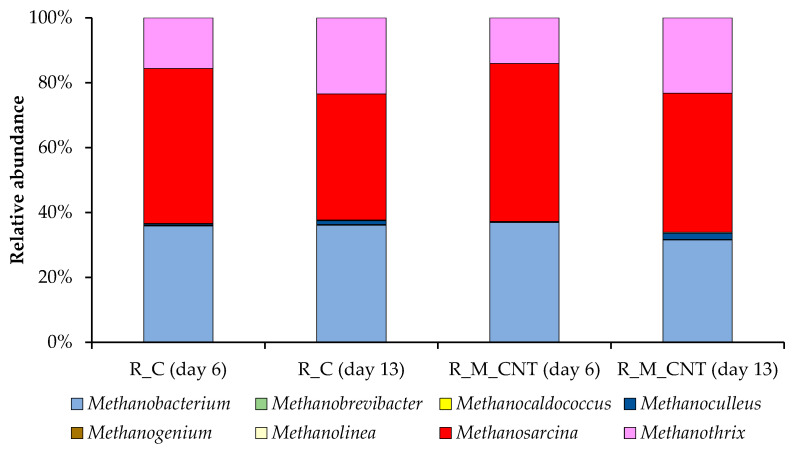
Taxonomic composition of methanogenic communities in the anaerobic reactors as determined by amplicon sequencing of *mcrA* genes.

The dominant archaeal classes observed in our systems were *Methanomicrobia* and *Methanobacteria* (phylum *Euryarchaeota*). The predominant archaeal genera observed in all samples from the R_C and R_M_CNT were *Methanosarcina*, *Methanothrix* (class *Methanomicrobia*), and *Methanobacterium* (class *Methanobacteria*). The relative abundance of each genus varied between reactors and sampling points (Figure 8). Our results are consistent with other data on the predominance of these microbial groups (responsible for the methanogenesis stage) during the anaerobic conversion of nitrogen-rich wastes [17,29,50].

Members of the mixotrophic genus *Methanosarcina* prevailed in both reactors on day 6 (48% and 49% of the overall methanogenic community in the R_C and R_M_CNT, respectively), whereas their level decreased during the operation of reactors. In contrast, the relative abundance of the acetoclastic genus *Methanothrix* increased in the R_C and R_M_CNT on day 13 (from 14–16% to 23–24%). This indicates that acetoclastic methanogenesis remained the major pathway of methanogenesis, despite the joint supplementation of the reactors with nano-Fe_3_O_4_ and CNTs. The relative abundance of representatives of the hydrogenotrophic genus *Methanobacterium* did not change strictly during the performance of the anaerobic digesters.

Methanogens of the genus *Methanosarcina* are more adapted to higher concentrations of organic acids and are more resistant compared to strict acetoclastic methanogens of the genus *Methanothrix* [51] and can also participate in the DIET mechanism [52]. Since the abundance of the genus *Methanothrix* increased in both reactors and considering that members of this this genus can accept electrons from bacterial partners via DIET for the reduction of carbon dioxide to methane [53], we assume its participation both in the process of the acetoclastic pathway of methanogenesis (in the R_C and R_M_CNT) and in DIET (in the R_M_CNT). It should be noted that the PCR data do not reflect absolute abundances.

Finally, this work noted the possibility of improving the conversion of the complex substrate both by creating conditions for reducing inhibition by metabolites and by providing favorable syntrophy and interspecies electron transfer between different microbial groups.

## 4. Conclusions

The addition of conductive nanomaterials (3.2 g L^−1^ of nano-Fe_3_O_4_ and 3.5 g L^−1^ of CNTs) to anaerobic reactors made it possible to enhance the consumption of produced VFAs and stimulate methanogenesis during the anaerobic digestion of chicken wastes. The joint addition of nano-Fe_3_O_4_ and CNTs to the anaerobic reactors increased the methane flow rate more efficiently compared to their separate use. The supplementation of anaerobic reactors with nano-Fe_3_O_4_ or CNTs promoted earlier consumption of acetate, butyrate, and finally propionate, and the joint addition of Fe_3_O_4_ and CNTs stimulated the more effective involvement of these VFAs in methanogenesis. In addition, anaerobic reactors supplemented with CNTs resulted in a lower level of TAN. The analysis of the microbial community noted the relationship between bacteria and archaea, confirming the possibility of a DIET mechanism. Members of the bacterial classes *Bacteroidia*, *Clostridia*, and *Actinobacteria* were detected at higher levels in the anaerobic reactors. Representatives of the genera *Methanosarcina*, *Methanobacterium*, and *Methanothrix* were mainly detected within the methanogenic communities in the anaerobic systems. Analysis of microbial community structures revealed that microbes capable of hydrogen interspecies transfer and direct interspecies electron transfer were enriched in the best-performing reactors. Due to their properties, nano-Fe_3_O_4_ and CNTs have a combined effect on the functioning and well-coordinated action of anaerobic communities. The results of the study will provide an approach to improving the efficiency of anaerobic conversion of chicken manure, in particular under conditions of inhibition by the accumulation of organic acids and ammonia. Finally, it is necessary to further investigate other potential materials that improve the anaerobic process as well as microorganisms with the potential for DIET.

## Figures and Tables

**Table 1:** Alpha diversity of microbial communities in the anaerobic reactors (analyzed on day 6 and day 13).

Sample	Bacteria				Methanogenic Archaea			
	OTUs	Chao1	Shannon	Simpson	OTUs	Chao1	Shannon	Simpson
R_C (day 6)	609	641	6.76	0.97	24	25	2.40	0.76
R_C (day 13)	661	673	7.10	0.98	25	27	2.46	0.77
R_M_CNT (day 6)	603	645	6.72	0.97	22	24	2.33	0.75
R_M_CNT (day 13)	624	664	6.84	0.97	27	32	2.47	0.77

## Data Availability

The data presented in this study are available on request from the corresponding author.
